# Cross-Talk Between Mesenchymal Stromal Cells (MSCs) and Endothelial Progenitor Cells (EPCs) in Bone Regeneration

**DOI:** 10.3389/fcell.2021.674084

**Published:** 2021-05-13

**Authors:** Cyril Bouland, Pierre Philippart, Didier Dequanter, Florent Corrillon, Isabelle Loeb, Dominique Bron, Laurence Lagneaux, Nathalie Meuleman

**Affiliations:** ^1^Department of Stomatology and Maxillofacial Surgery, Saint-Pierre Hospital, Brussels, Belgium; ^2^Laboratory of Clinical Cell Therapy, Jules Bordet Institute, Université Libre de Bruxelles, Brussels, Belgium; ^3^Faculty of Medicine, Université Libre de Bruxelles, Brussels, Belgium; ^4^Department of Stomatology and Maxillofacial Surgery, IRIS South Hospital, Brussels, Belgium; ^5^Department of Hematology, Jules Bordet Institute, Université Libre de Bruxelles, Brussels, Belgium

**Keywords:** bone regeneration, endothelial progenitor cell (EPC), mesenchymal stromal cell (MSC), cross-talk, review

## Abstract

Bone regeneration is a complex, well-orchestrated process based on the interactions between osteogenesis and angiogenesis, observed in both physiological and pathological situations. However, specific conditions (e.g., bone regeneration in large quantity, immunocompromised regenerative process) require additional support. Tissue engineering offers novel strategies. Bone regeneration requires a cell source, a matrix, growth factors and mechanical stimulation. Regenerative cells, endowed with proliferation and differentiation capacities, aim to recover, maintain, and improve bone functions. Vascularization is mandatory for bone formation, skeletal development, and different osseointegration processes. The latter delivers nutrients, growth factors, oxygen, minerals, etc. The development of mesenchymal stromal cells (MSCs) and endothelial progenitor cells (EPCs) cocultures has shown synergy between the two cell populations. The phenomena of osteogenesis and angiogenesis are intimately intertwined. Thus, cells of the endothelial line indirectly foster osteogenesis, and conversely, MSCs promote angiogenesis through different interaction mechanisms. In addition, various studies have highlighted the importance of the microenvironment via the release of extracellular vesicles (EVs). These EVs stimulate bone regeneration and angiogenesis. In this review, we describe (1) the phenomenon of bone regeneration by different sources of MSCs. We assess (2) the input of EPCs in coculture in bone regeneration and describe their contribution to the osteogenic potential of MSCs. We discuss (3) the interaction mechanisms between MSCs and EPCs in the context of osteogenesis: direct or indirect contact, production of growth factors, and the importance of the microenvironment via the release of EVs.

## Introduction

Bone regeneration is a complex, well-orchestrated process based on the interactions between osteogenesis and angiogenesis (Fröhlich, 2019), observed in both physiological and pathological situations. However, spontaneous bone regeneration could be overwhelmed in several complex clinical conditions (e.g., large bone defect subsequent to trauma, infection, tumor resection and skeletal abnormalities, or compromised regenerative process due to avascular necrosis, atrophic non-unions and osteoporosis) (Dimitriou et al., 2011). Tissue-engineering offers new approaches to foster bone regeneration (Bouland et al., 2020). Bone regeneration requires four elements: a cell source, a scaffold, tissue-inducing factors (signaling factors) and mechanical stimulation (Perez et al., 2018).

Since 2001, when the first three patients were successfully treated for bone defects with expanded autologous bone marrow mesenchymal stromal cells (BM-MSCs) (Quarto et al., 2001), numerous studies have been conducted (Watson et al., 2014; Perez et al., 2018). Different mesenchymal stromal cells (MSCs) sources have been studied in bone tissue engineering in both clinical and preclinical settings (Perez et al., 2018). However, successful bone regeneration relies on vascularization (Rouwkema and Khademhosseini, 2016; Filipowska et al., 2017). Indeed, insufficient vascularization during the initial phase, after *in vivo* implantation, could lead to insufficient cell integration and death (Pang et al., 2013; Grosso et al., 2017; Fröhlich, 2019). A functionally perfused vascular network also mediates the recruitment of osteoprogenitors, hematopoietic stem cells (HSCs) and immune cells, playing a significant role in tissue regeneration and remodeling (Grosso et al., 2017). Furthermore, vascularization participates in structural and functional reconstruction (Pang et al., 2013).

Different strategies have been undertaken to introduce the vasculature into tissue-engineered constructs, including scaffolds, growth factors, *in vitro* or *in vivo* graft prevascularization and coculture (Liu et al., 2015). MSCs and endothelial progenitor cells (EPCs) coculture aims to obtain a synergistic effect in terms of angiogenesis and bone regeneration (Sun et al., 2016). EPCs have been shown by many researchers to be effective in cell-based therapies, improving vascularization for a variety of applications (Atesok et al., 2010). EPCs stimulate angiogenesis and osteogenesis by the secretion of trophic factors (Pang et al., 2013; Jia et al., 2019).

In addition, the therapeutic potential of MSCs, through the production of trophic factors, could modulate the host tissue microenvironment and subsequently facilitate the regenerative process (Li et al., 2012). Recently, Jia et al. (2019) outlined the importance of the microenvironment, particularly extracellular vesicles (EVs), which are considered an important component of cellular paracrine secretion. EVs secreted by EPCs markedly accelerate bone regeneration by stimulating angiogenesis (Jia et al., 2019). In this review, we will first describe the different MSCs sources used to generate bone and analyze relevant clinical studies. We will evaluate the contribution of MSC/EPC coculture to bone regeneration. Finally, we will describe herein the different interactions between MSCs and EPCs. These elements will determine the benefits of MSC/EPC coculture in bone regeneration and the related interactions between these two cell populations.

## Application of MSCs in Bone Regenerative Medicine

**FIGURE 1:**
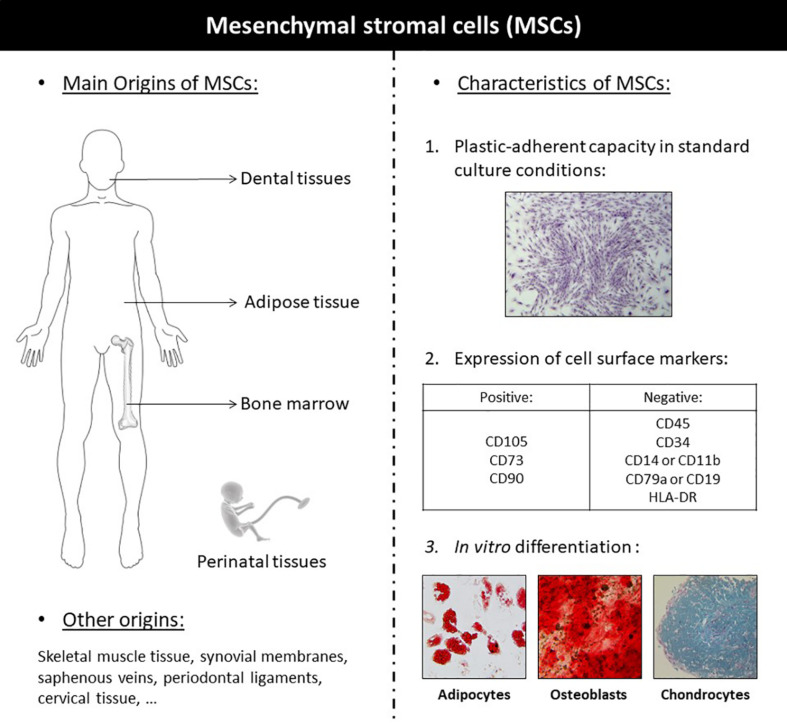
Definition and illustration of the different sources of mesenchymal stromal cells.

Mesenchymal stromal cells are multipotent adult stem cells exhibiting multilineage differentiation potential (Squillaro et al., 2016). MSCs are defined by the three following criteria: adherence to plastic, specific surface antigen (Ag) expression, and multipotent differentiation potential, according to the International Society for Cellular Therapy (ISCT) (Figure 1; Dominici et al., 2006). They exhibit great differentiation potential into many different types of tissue lineages, including bone (osteoblasts), cartilage (chondrocytes), muscle (myocytes), and fat (adipocytes). MSCs have been isolated from multiple tissues: bone marrow (BM), skeletal muscle tissue, adipose tissue (AT), synovial membranes, saphenous veins, dental pulp, periodontal ligaments, cervical tissue, Wharton’s jelly, umbilical cords, umbilical cord blood, amniotic fluid, placentae, etc. (Squillaro et al., 2016). In 1995, the first MSCs clinical study used autologous, culture-expanded BM-MSCs in patients with hematological malignancies, demonstrating safety with no reports of adverse events (Koç et al., 2000). Since this first trial, over 3,000 patients enrolled in more than 100 clinical trials have met safety endpoints, with no serious adverse events reported to date (Watson et al., 2014). Since then, MSCs have been studied in numerous clinical studies for representative diseases, including organ transplantation, diabetes, inflammatory, hematological, cardiovascular, auto-immune, cartilage, and bone disease (Squillaro et al., 2016). Clinical studies promoted non-union fracture healing (Hernigou et al., 2015), accelerated fracture healing (Kim et al., 2009) or bone regeneration in bone tissue defect (Pavan Kumar et al., 2016) and other pathological conditions (Manimaran et al., 2014, 2016; Bouland et al., 2020). The clinical applications of the different sources of MSCs are detailed in Table 1.

**TABLE 1:** Summary of the Clinical studies using mesenchymal stromal cells for bone regeneration.

References	Cell type	Procedure	Patient’s group	Follow-up (average)	Main results
Umemura et al., 2006	Autologous BM-MNCs	Implantation of 1.1 × 10^9^ BM-MNCs in the gastrocnemius muscle around the fractured bone	Tibial non-union with compartment syndrome (*N* = 1)	6 months	After treatment: • Marked formation of collateral vessels and slight increase of the callus at the fracture site (4 PO weeks) • Complete fracture healing (6 PO months)
Liebergall et al., 2013	Autologous Purified CD105^+^ BM cells	Percutaneous injection of 1 × 10^8^ purified CD105^+^ BM cells mixed with PRP and DBM into the fracture site	Extra-articular distal tibial fracture [Study group (*N* = 12); Ctrl group (*N* = 12)]	12 months	Implantation of CD105^+^ BM cells into the fracture site significantly reduce time to union
Hernigou et al., 2015	Autologous BMAC	Percutaneous injection of 27.3 ± 14.6 × 10^6^ cells/mL (mean) BMAC	Anckle non-union in diabetic patients [Study group (*N* = 86); Ctrl group (*N* = 86)]	6 months	Treatment with BMAC promoted non-union healing in 82.1% with a low number of complications. • Treatment with iliac bone graft promoted non-union healing in 62.3%, major complications were observed: 5 amputations, 11 osteonecroses of the fracture wound edge and 17 infections.
Hernigou and Beaujean, 2002	Autologous BMAC	Core decompression and injection of 16.4 × 10^6^/mL BMAC	ONFH (*N* = 116; 189 hips)	7 years	After treatment: • Hip replacement: 18% (34/189) (Mean: 26 months) • Stage I and II (before collapse) patients: hip replacement 6% (9/145) • Stage III and IV (after collapse) patients: hip replacement 57% (25/44) • Better outcome for a greater number of transplanted progenitor cells
Hernigou et al., 2009	Autologous BMAC	Core decompression and injection of 29 × 10^6^/mL BMAC	ONFH stage I and II (*N* = 342; 534 hips)	13 years	After treatment: • Hip replacement: 17.6% 94/534 • Stage I (before collapse): resolution of the ONFH (69/534) • Stage I and II (before collapse) patients: ONFH improvement: (371/534)
Cai et al., 2014	Autologous BM-MNCs and allogeneic UC-MSCs	Arterial perfusion of 60,7 (±11.5) × 10^6^/kg BM-MNCs and 1.0 (±0.1) × 10^6^/kg umbilical cord mesenchymal stromal cells (UC-MSCs)	ONFH (*N* = 30; 49 hips)	16.9 months	After treatment: • Relief of hip pain (93.3%), joint function improvement (86.7%), and extended walking distances (86.7%) • Harris hip scores significantly increased (3, 6, and 12- PO months). • MI: bone lesions improvement (89.7%) (44/49)
Cella et al., 2011	Autologous BM-MNCs	Implantation of 426 × 10^6^ BM-MNCs and PRP in a fibrin sponge after BRONJ surgical debridement	Stage III BRONJ (*N* = 1)	30 months	After treatment: • Symptoms resolution and progressive mucosal healing (2 PO weeks) • MI: bone regeneration (15 PO months) • Uneventful follow-up • No recurrence of MRONJ
Pavan Kumar et al., 2016	Autologous BM-MNCs	Local transplantation of 2.56 × 10^8^ BM-MNCs [Iliac bone (*N* = 20)] or 1.74 × 10^7^ BM-MNC [mandible (*N* = 10)]	Hard tissue defect (*N* = 15): cystic lesions *N* = 6 post-surgical alveolar defects *N* = 4 peri implant defects *N* = 3 alveolar clefts *N* = 2 Soft tissue lesion (*N* = 15): leukoplakia and lichen planus *N* = 6 OSMF *N* = 7 post traumatic soft tissue loss *N* = 2	6 months	Hard tissue defect: • MI: bone regeneration, bridging the defect (3 PO months) • MI: Regenerated bone similar to native bone (6 PO months) Soft tissue lesion: • OSMF (7): adequate clinical mouth opening, reduction in burning sensation and blanching of mucosa • Leukoplakia and lichen planus (6): good clinical improvement. • Post traumatic soft tissue defects (2): good clinical improvement.
Quarto et al., 2001	Autologous expanded BM-MSCs	Local application of BM-MSCs on a macroporous HA scaffold in association with external fixation for mechanical stability	Large bone defect (4→7 cm) (*N* = 3)	15→27 months	After treatment: • Limb function recovered • Mi: abundant callus formation along the implants, good integration at the interfaces with the host bones (2 PO months) • Uneventful follow-up
Marcacci et al., 2007	Autologous expanded BM-MSCs	Local application of BM-MSCs on a macroporous HA scaffold in association with external fixation for mechanical stability	Large bone defect (4→7 cm) (*N* = 4)	1.25→7 years	After treatment: • Complete fusion between the implant and the host bone (5→7 PO months) • Good implant integration (up to 7 PO years) • Uneventful follow-up
Morishita et al., 2006	Autologous cultured BM osteoprogenitors	Implantation of autologous cultured BM osteoprogenitors on HA ceramics in the bone defects after tumor curettage	Benign bone tumors (*N* = 3): Aneurysmal bone cyst (*N* = 1) Giant cell tumor (*N* = 1) Fibrous dysplasia (*n* = 1)	29→43 months	After treatment: • MI: Incorporation of the cultured cells into the host bone (3 PO months). • Uneventful follow-up
Kitoh et al., 2009	Autologous expanded BM-MSCs	Injection of 1.23 ± 0.62 10^7^ (femur) or 1.45 ± 0.56 10^7^ (tibia) BM-MSCs and PRP in the site of distraction	Femoral and tibial lengthening [study group (*N* = 28, 51 bones (23 femora and 28 tibiae)) Ctrl group (60 bones without therapy)]	>3 months	The healing index was significantly lower in the study group • Femoral lengthening showed significantly faster healing than tibial lengthening
Kim et al., 2009	Autologous cultured osteoblasts	Injection of 1.2 10^7/^0.4 ml mixed with fibrin (ratio 1/1) in the fracture area	Long-bone fractures (*N* = 64) [Study group (*N* = 32) Ctrl group (*N* = 32)]	1→2 months	Autologous cultured osteoblast injection significantly accelerates fracture healing • No complications observed
Zhao et al., 2012	Autologous expanded BM-MSCs	Core decompression and implantation of 2.6 × 10^6^ BM-MSCs in the femoral head	ONFH early stage (*N* = 100) [Study group (*N* = 50, 53 hips); Ctrl group (*N* = 50, 51 hips)]	5 years	Treatment with core decompression and BM-MSCs, significant improvement the Harris hip score and decreased necrotic bone volume; ONFH progression: 2/53, Subsequent vascularized bone grafting (2/2) • Treatment core decompression: ONFH progression: 10/44, subsequent vascularized bone grafting (5/10) or hip replacement (5/10)
Gómez-Barrena et al., 2019	Autologous BM-MSCs	Application of 100–200 × 10^6^ BM-MSCs mixed with BCP surgically delivered in the non-union site	Fracture non-union (*N* = 28)	1 year	Display feasibility and safety of BCP and BM-MSCs in non-union fractures • MI: non-union healing in 26/28 patients (12 PO months) • AP: bone formation surrounding the BCP granules
Lendeckel et al., 2004	Autologous SVF	Application of 295 × 10^6^ SVF mixed with autologous fibrin glue in addition to bone grafting	Calvarial defect (*N* = 1)	3 months	After treatment: • MI: new bone formation and near complete calvarial continuity (3 PO months) • Uneventful follow-up
Mesimäki et al., 2009	Autologous expanded AT-MSCs	Application of 13 × 106 AT-MSCs mixed with βTCP and rhBMP-2 in the left rectus muscle. Rectus abdominis free flap (containing the AT-MSCs) raised to reconstruct the bone defect (±10 months later)	Keratocyst (*N* = 1)	1 year	After treatment: • MI: bone formation • Dental implants placed in the grafted site • Uneventful follow-up • No recurrence of the keratocyst.
Thesleff et al., 2011	Autologous expanded AT-MSCs	Application of 15 × 10^6^ AT-MSCs mixed with βTCP in the bone defect in association with a mesh to reconstruct the bone defect	Calvarial defect (*N* = 4)	3 months (*N* = 2) 1 year (*N* = 2)	After treatment: • good clinical outcome • Medical imaging: bone regeneration • No complication observed.
Sándor et al., 2013	Autologous expanded AT-MSCs	Application of 10^6^ AT-MSCs mixed with βTCP and rhBMP-2 in association with a mesh to reconstruct the bone defect	Ameloblastoma (*N* = 1)	3 years	After treatment: • MI: bone formation • No complication observed • Dental implants placed in the grafted site. • No recurrence of the ameloblastoma
Sándor et al., 2014	Autologous expanded AT-MSCs	Application of 2.8→16 × 10^6^ AT-MSCs mixed with βTCP (*N* = 10) or BAG (*N* = 3) to reconstruct the bone defect	Cranio-maxillofacial defects (=13): Frontal sinus (*N* = 3) Cranial bone (*N* = 5) Mandible (*N* = 3) Nasal septum (*N* = 2)	37 months	After treatment: • Successful integration of the construct to the surrounding skeleton (10/13) • Two cases of cranial defect needed a second procedure • One case of septal perforation failed
Khalpey et al., 2015	Autologous uncultured SVF	Local injection: 100 × 10^6^ SVF and intravenous injection: 200 × 10^6^ SVF in association with conventional plating system	Sternal non-union with bone defect (*N* = 1)	months	After treatment: • Stabilized sternum with nearly no pain and normal exercise tolerance • MI: fracture healing and closure of areas of non-union (3, 6 PO months) • Uneventful follow-up
Saxer et al., 2016	Autologous uncultured SVF	Application of SVF mixed with Ceramic granules and fibrin hydrogel in the void space of the fracture zone upon ORIF	Displaced low-energy fractures of the proximal humerus (*N* = 8)	12 months	SVF, without expansion or exogenous priming, can spontaneously form bone tissue and vessel structures within a fracture-microenvironment After treatment: • Pain-free range of movement sufficient (within a year) • AP: *de novo* bone formation (5/6) • Uneventful follow-up
Prins et al., 2016	Autologous uncultured SVF	Local application of SVF mixed with BCP (*N* = 5) or βTCP (*n* = 5)	MSFE (*N* = 10)	>2.5 years	Display feasibility, safety, and efficiency of SVF seeded on bone substitutes for MSFE. • AP: Bone and osteoid percentages were higher in the SVF group, independent of the bone substitute • No adverse effects
Farré-Guash et al., 2018	Autologous uncultured SVF	Local application of SVF mixed with BCP (*N* = 5) or βTCP (*n* = 5)	MSFE (*N* = 10)		Display feasibility, safety, and efficiency of SVF seeded on bone substitutes for MSFE • Pro-angiogenic effect of SVF • AP: correlation between bone percentages and blood vessel formation, bone percentage higher in the SVF group in the cranial area. • No adverse effects
Bouland et al., 2020	Autologous uncultured SVF	Local application of, 48.1 × 10^6^ SVF injected in L-PRF after bone debridement (Case 1) Local application 20.8 × 10^6^ SVF injected in L- PRF after bone debridement (Case 2)	MRONJ (*N* = 2)	2 years	Case 1: After treatment: • Symptoms resolution and mucosal closure (2 PO weeks) • MI: bone formation (6, 12, 18 PO months) • Uneventful follow-up • No recurrence of MRONJ Case 2: After treatment: • Symptoms resolution and mucosal closure (2 PO weeks) • MI: bone formation (6,12,18 PO months) • Uneventful follow-up • No recurrence of MRONJ
Dilogo et al., 2017	Allogeneic expanded UC-MSCs	Application of 50 × 10^6^ UC-MSCs mixed with HA and BMP-2 and mechanical stimulation (Masquelet technique)	Infected non-union Femoral fracture with a bone defect (*N* = 1)	12 months	After treatment: • Clinical union, walking with a crutch, no pain during the walk and clinical leg length discrepancy (2 cm). LEFS improvement at 30% (6 PO months) • Full weight bearing walk without pain, with an improved LEFS and no leg length discrepancy (11 PO months) • MI: progressive bone formation (1, 3, 6, 9, 12 PO months) • Uneventful follow-up
Rahyussalim et al., 2020	Allogeneic expanded UC-MSCs	Application of 20 × 10^6^ UC-MSCs mixed with HA after surgical debridement to reconstruct the bone defect	Vertebral body bone defect (*N* = 1)	6 months	After treatment: • Walking without pain (3 PO months). • Uneventful follow-up • No signs of neoplasm formation, no significant bone deformation or spinal cord compression.
Manimaran et al., 2014	Autologous expanded DP-MSCs and uncultured BMAC	Application of BMAC mixed with βTCP and HA after curettage of the necrotic bone (Case 1) Application of DPMSC mixed with βTCP and PRP after curettage of the necrotic bone (Case 2)	ORN (*N* = 2)	Case 1: 2 years Case 2: 6 months	Case 1: After treatment: • gradual pain reduction and no intraoral discharge (2 PO months) • MI: bone formation (2 PO months) and resolution of the suspected fracture line (6 PO months) • Follow-up uneventful • No recurrence of the ORN Case 2: After treatment: • MI: bone formation (2, 6 PO months) • Follow-up uneventful • No recurrence of the ORN
Manimaran et al., 2016	Autologous expanded DP-MSCs and uncultured SVF	Application of 20 × 10^6^ DPMSC and 45 × 10^6^ SVF mixed with PRP and βTCP, covered with PRF in association with a mesh to reconstruct the bone defect after resection of the ameloblastoma	Ameloblastoma (*N* = 1)	1.5 years	After treatment: • MI: bone formation (10 PO months) • Follow-up uneventful • No recurrence of the ameloblastoma

*βTCP, β-tricalcium phosphate; AP, anatomopathology; BAG, bioactive glass; BCP, biphasic calcium phosphate; BMAC, bone marrow aspirate concentrate; BM-MSC, bone marrow mesenchymal stromal cell; BM-MNC, bone marrow mononuclear cell; BRONJ, bisphosphonate-related osteonecrosis of the jaw; CT, computed tomography; DBM, demineralized bone matrix; HA, hydroxyapatite; LEFS, lower extremity function scale; MI, medical imaging; MSFE, maxillary sinus floor elevation; MRONJ, medication-related osteonecrosis of the jaw; ONFH, osteonecrosis of the femoral head; ORIF, open reduction internal fixation; ORN, osteoradionecrosis; OSMF, oral sub-mucous fibrosis; rhBMP-2: PRP, platelet-rich plasma; L-PRF, leukocyte-platelet rich fibrin.*

### Bone Marrow

The BM is the first studied source of MSCs. Several MSC-based cell therapy modalities have been developed with or without cell culturing and with or without matrix. BM-MSCs have significant bone repair and regeneration potential. However, BM-MSC *in vitro* culture requires time, expensive manufacturing costs, and good manufacturing practice (GMP) facilities, and it presents a contamination risk. These factors render the cells requiring *in vitro* amplification unsuitable for clinical use. Moreover, during culture, BM-MSCs are exposed to hyperoxic conditions, leading to elevated levels of intracellular reactive oxygen species (ROS) production. Robust assays are required to monitor biological changes that could reduce the efficacy of cell-based therapy. Relative to BM-MSCs, bone marrow mononuclear cells (BM-MNCs) are a heterogeneous cell population that includes MSCs, EPCs, HSCs, and lymphocytes. BM-MNCs can be directly applied bypassing the *in vitro* expansion process. This can save time, expense and avoid the declining differentiation and migration ability caused by *in vitro* culture, as well as contamination risk and other uncertain factors, such as genetic aberrations not associated with malignant transformation or *in vitro* transformed phenotype (Capelli et al., 2014; Du et al., 2020). Recently, Du et al. (2020) compared and observed that fresh BM-MNCs promote more bone regeneration than *in vitro* expanded BM-MSCs in an animal model. Moreover, the grafts containing the BM-MNCs were better mineralized, with collagen arrangement and microbiomechanical properties similar to those of native tibia (Du et al., 2020). Furthermore, Sun et al. (2009) highlighted that BM-MNCs increase both neovascularization and bone regeneration in an animal model of osteonecrosis of the hip (ONFH) after core decompression.

In 2006, a first patient suffering from compartment syndrome and bone non-union was treated by autologous BM-MNC implantation for therapeutic angiogenesis and subsequent bone regeneration. Four weeks later, angiography showed a marked increase in collateral vessels surrounding the tibial fracture, and union was completed 6 months later. The authors suggested that the increased blood flow helped maintain the peripheral tissue structural and functional integrity and subsequently facilitated bone regeneration (Umemura et al., 2006). Few years later, in a randomized controlled clinical study, Liebergall et al. (2013) assessed that autologous fresh purified CD105^+^ BM cells (10^8^) injected into a reduced fracture together with platelet-rich plasma (PRP) and allograft demineralized bone matrix significantly reduce time to union. Diabetic patients treated for non-union ankle fractures with BM-MSCs showed a higher rate of successful unions and far fewer complications compared to diabetics receiving iliac crest bone graft as a standard of care. Treatment with percutaneous bone marrow aspirate concentrate (BMAC) promoted non-union healing in 70/86 diabetic patients (82.1%) with a low percentage of complications. In comparison, treatment with bone iliac crest autografts promoted non-union healing in only 53/86 diabetic patients (62.3%). Major complications were assessed in the control group: five amputations, 11 osteonecroses of the fracture wound edge and 17 infections (Hernigou et al., 2015).

In parallel, Hernigou and Beaujean (2002) and Hernigou et al. (2009) published several papers related to ONFH treatment with autologous BMAC with good results. In 2002, 116 patients suffering from ONFH (189 hips) were treated with core decompression and BMAC (Hernigou and Beaujean, 2002). During the follow-up (5–11 years), only 18% (34) of the hips and 20% (22) of the patients, required total hip replacement at a mean of 26 months after decompression and autologous BMAC grafting. In 2009, 342 patients suffering from stage I and II ONFH (534 hips) were treated with core decompression and autologous bone marrow grafting (Hernigou et al., 2009). The patients were followed up from eight to 18 years. Hip replacement was performed in 94/534 cases (17.6%) after collapse. The authors suggested that this procedure should be advised for symptomatic ONFH without collapse. Cai et al. (2014) treated also patients suffering from ONFH. However, the authors injected an association of autologous BM-MNCs and allogeneic umbilical cord mesenchymal stromal cells (UC-MSCs) by arterial perfusion (Cai et al., 2014). The patients were followed for 16.9 months. The Harris scores evaluating pain relief, joint function, walking distance, and image changes were significantly improved (*P* < 0.05) 3, 6, and 12 months after the procedure. Twenty-eight patients (93.3%) showed hip pain relief, 26 (86.7%) showed improved joint function, and 26 patients (86.7%) benefited from an extended walking distance.

Cella et al. (2011) treated a stage III bisphosphonate-related osteonecrosis of the jaw (BRONJ) with autologous BM-MNCs and PRP. Fifteen months after the procedure, medical imaging assessed bone regeneration and concentric ossification. Soft and hard tissue were assessed during the 30-month uneventful follow-up. Manimaran et al. (2014) treated a case of osteoradionecrosis (ORN) with the association of autologous BMAC, beta-tricalcium phosphate (βTCP) and hydroxyapatite (HA). The treated patient stayed asymptomatic during the follow-up, and moreover, a complete bone remodeling was noticed after 1 year. Pavan Kumar et al. (2016) successfully treated 30 patients with soft or hard tissue defects in the oral and maxillofacial areas with autologous BM-MNCs and observed beneficial effects in bone regeneration and soft tissue wound healing.

Notwithstanding, the regenerative potential of therapies based on expanded BM-MSCs is being tested clinically for the treatment of bone defects, fractures and osteonecrosis (Grayson et al., 2015). Quarto et al. (2001) were the first to report the use of cultured BM-MSCs combined intraoperatively with HA scaffolds to fill large bone defects in three patients. Then, the study of Marcacci et al. (2007) highlighted the long-term durability of bone regeneration achieved by bone engineering. The authors confirmed the use of culture-expanded osteoprogenitor cells in conjunction with porous bioceramics to improve the repair of critical-sized long bone defects. This technique was successfully used by Morishita et al. (2006) in three patients with benign bone tumors. The use of cultured BM-MSCs in association with PRP improved the healing index during lengthening of 51 femurs or tibias compared to 60 controls (Kitoh et al., 2009). A multicenter, randomized, clinical trial reported that autologous cultured osteoblast injection accelerates significantly fracture healing (Kim et al., 2009).

In a randomized trial of 100 patients, Zhao et al. (2012) treated 50 patients suffering from ONFH with culture-expanded autologous BM-MSCs. The authors followed the patients for 5 years. Only two of the 53 BM-MSC-treated hips progressed and underwent vascularized bone grafting. BM-MSCs treatment significantly improved the Harris hip score and decreased the volume of the necrotic lesion. In a European multicenter clinical trial, the surgical implantation of GMP-expanding BM-MSCs in combination with bioceramic granules was feasible and safe for the treatment of fracture non-union and ONFH. Moreover, 26/28 treated patients were radiologically healed at 1 year (Gómez-Barrena et al., 2019).

### Adipose Tissue

The AT is another studied source of MSCs. Described first by Zuk et al. (2001) adipose tissue-mesenchymal stromal cells (AT-MSCs) are easily harvested by lipoaspiration in large quantities with minimal discomfort. AT contains a higher stromal cell to volume ratio than BM (Zuk et al., 2002; Mesimäki et al., 2009). The Stromal vascular fraction (SVF) processed from excised adipose tissue, by mechanical or enzymatic isolation, is a heterogeneous cell population containing not only adipose stromal and hematopoietic stem and progenitor cells but also endothelial cells, erythrocytes, fibroblasts, lymphocytes, monocytes/macrophages and pericytes (Zuk et al., 2001; Bourin et al., 2013). AT-MSCs exhibit similar properties to BM-MSCs. However, numerous features distinguish these two cell populations. AT-MSCs display stronger adipogenic, similar chondrogenic and lower osteogenic differentiation potential (Xu et al., 2017). Moreover, AT-MSCs display significant proangiogenic potential *in vivo* (Rehman et al., 2004). AT-MSCs may play an important role in bone tissue regeneration, and their secretome includes several endocrine and pro-angiogenic factors able to induce bone activity (Paduano et al., 2017).

Lendeckel et al. (2004) and Mesimäki et al. (2009) observed bone regeneration in a clinical setting. Lendeckel et al. (2004) treated a patient suffering from a calvarial defect with a combination of macropore sheets, cancellous bone, adipose-derived mononuclear cells (containing AT-MSCs) and autologous fibrin glue. The computed tomography (CT) scan performed 3 months after the operation showed marked ossification in the defect areas (Lendeckel et al., 2004). Later, Mesimäki et al. (2009) reconstructed a maxillary defect with autologous expanded AT-MSCs and recombinant human (rh) BMP-2 and βTCP granules inside a muscle-free flap. After 36 months of follow-up, the dental implants were osteointegrated, and the bony defect was reconstructed (Mesimäki et al., 2009). Thesleff et al. (2011) assessed bone regeneration using the combination of AT-MSCs and βTCP in four patients with critical-size calvarial defects.

Sándor et al. (2013, 2014) successively published two papers tracking their experience. First, the authors achieved a 10-cm anterior mandibular reconstruction with expanded AT-MSCs, βTCP and rhBMP-2. Histomorphometric analysis showed that the recovered bone core consisted of woven bone (36.7%), osteoids (32.4%), fibrous tissue (23.3%), and residual scaffolds (8.6%) (Sándor et al., 2013). Thereafter, 13 maxillofacial cases were treated with either bioactive glass or βTCP scaffolds seeded with expanded AT-MSCs and, in some cases, with the addition of rhBMP-2. Follow-up time ranged from 12 to 52 months. Successful integration of the construct into the surrounding skeleton was noted in 10 of the 13 cases (Sándor et al., 2014).

AT-MSCs are isolated as part of the aqueous fraction derived from enzymatic digestion of AT. This fraction, known as the SVF, contains AT-MSCs, EPCs, endothelial cells (ECs), macrophages, smooth muscle cells, lymphocytes, pericytes, and adipocytes, among others. SVF displays similar properties to AT-MSCs, such as immunomodulation, anti-inflammation, and angiogenesis, but the distinctive, heterogeneous cellular composition of SVF may be responsible for the better therapeutic outcome observed in different animal studies (Bora and Majumdar, 2017).

Jurgens et al. (2013) noticed in an animal model that expanded AT-MSCs and fresh SVF exhibited similar osteogenic potential. Furthermore, fresh SVF can induce neovascularization through dynamic reassembly of blood endothelial cells (Koh et al., 2011).

In 2015, the first sternum reconstruction using SVF in conjunction with plating techniques was performed. Symptoms improved commensurate with healed areas of non-union 3 months after the operation and still maintained 3 months later. The association of the different cell populations contained in the SVF was suspected to act synergistically (Khalpey et al., 2015). In 2016, eight patients with low-energy proximal humeral fractures were treated with fresh autologous SVF loaded onto ceramic granules within fibrin gel along with standard open reduction and internal fixation (Saxer et al., 2016). Interestingly, the authors suggested that SVF, without expansion or exogenous priming, could spontaneously form bone tissue and vessel structures within a fracture microenvironment. Prins et al. (2016) and Farré-Guash et al. (2018) evaluated bone regeneration for maxillary sinus floor elevation (MSFE) with SVF seeded on either βTCP or biphasic calcium phosphate (BCP) carriers. Bone and osteoid percentages were higher, supported by more mature angiogenesis in the core of the grafted material, in study biopsies (SVF supplemented) than in control biopsies, particularly in βTCP-treated patients (Prins et al., 2016). Furthermore, the vessel distribution was more homogeneous in the study biopsies (Farré-Guash et al., 2018).

In 2016, a patient with an ameloblastoma was cared for with a combination of expanded autologous dental pulp MSCs (DP-MSCs), SVF, PRP, PRF, and βTCP. Bone regeneration was confirmed through medical imaging. The authors observed no recurrence during the one and a half year follow-ups (Manimaran et al., 2016). Two patients suffering from medication-related osteonecrosis of the jaw (MRONJ) were treated with a combination of L-PRF and fresh SVF in a one-step procedure. The follow-up was uneventful, and based on CT scans during the 18 months of follow-up, bone regeneration was noticed (Bouland et al., 2020).

### Perinatal Derivatives

Perinatal derivatives (PnD) consist of birth-associated tissues, cells isolated thereof, and the factors secreted (Silini et al., 2019). The term “perinatal” refers to birth-associated tissues obtained from term placentas and fetal annexes. It refers to amniotic/amnionic membrane, chorionic membrane, chorionic villi, umbilical cord (including Wharton’s jelly), basal plate (including maternal and fetal cells), and amniotic fluid. The term “derivatives” consists of the isolated cells from placental tissues and the factors released by these cells. All the perinatal derivatives-mesenchymal stromal cells (PnD-MSC) present several advantages: a painless and non-invasive harvesting procedure, a low risk of infection and low immunogenicity, and sufficient experience for their clinical application. Furthermore, these MSCs meet the requirements of ethical issues and are very effective at differentiating into osteoblasts (Jäger et al., 2009; Ding et al., 2015). Moreover, UC-MSCs are less differentiated (Zajdel et al., 2017), have higher proliferative potential than adult MSCs (Subramanian et al., 2015) and have higher clonogenic abilities (Laroye et al., 2019) and a slower aging rate (Batsali et al., 2017). Chen et al. (2013) compared the osteogenic properties of BM-MSCs and UC-MSCs *in vivo*. The authors noticed that similar mineral densities and amounts of bone and vessels were assessed after seeding BM-MSCs or UC-MSCs in an animal calvarial defect model. Hao et al. (2014) observed that intrabone marrow injection of UC-MSCs also promoted new bone formation in a peri-implant defect animal model after immediate implantation. In addition, increased expression of osteogenic genes [alkaline phosphatase (ALP), type 1 collagen and osteocalcin] and a larger trabecular bone area in an osteoporotic animal model were demonstrated after external induction of UC-MSCs (Hendrijantini et al., 2019). In 2017, the first patient suffering from an infected non-union right femoral shaft fracture with significant bone loss was successfully treated with a combination of allogeneic UC-MSCs, bone morphogenetic protein-2 (BMP-2), hydroxyapatite (HA), and mechanical stabilization using the Masquelet technique (Dilogo et al., 2017). Three years later, a case of vertebral defect was successfully treated with expanded UC-MSCs and HA (Rahyussalim et al., 2020).

### Dental Tissues

Recently, the study spectrum of dental tissue MSCs has been broadened to bone regeneration. Gronthos et al. (2000) isolated dental pulp MSCs (DP-MSCs) in 2000. This source has the advantage of being easy to harvest (Huang et al., 2009). The DP-MSCs and BM-MSCs have comparable properties. Laino et al. (2005) obtained *in vitro* living autologous fibrous bone (LAB) tissue after DP-MSC differentiation. After transplantation into immunocompromised rats, lamellar bone with osteocytes within the bone and osteocytes surrounding the trabecula were highlighted. Thereafter, d’Aquino et al. (2007) obtained vascularized bone tissue for the first time. The authors noticed that DP-MSCs synergically differentiate into osteoblasts and endotheliocytes and that flk-1 exerts a pivotal role in coupling osteoblast and endotheliocyte differentiation (d’Aquino et al., 2007). In parallel, MSCs from other dental tissues were identified: exfoliated deciduous tooth stem cells (SHEDs) (Miura et al., 2003), periodontal ligament stem cells (PDLSCs) (Seo et al., 2004), apical papilla stem cells (APSCs) (Sonoyama et al., 2006), dental follicle stem cells (DFSCs) (Morsczeck et al., 2005), and gingival mesenchymal stromal cells (GMSCs) (Mitrano et al., 2010). The relationship between different dental tissue MSCs remains unclear (Huang et al., 2009). All dental tissue MSCs have a similar capacity to differentiate into other cell lineages but are not identical to BM-MSCs (Huang et al., 2009). Different studies noted that SHEDs, DP-MSCs and BM-MSCs have similar osteogenic properties *in vivo* (Nakajima et al., 2018; Lee et al., 2019). Nevertheless, Lee et al. (2019) observed that BM-MSCs had higher osteogenic differentiation ability *in vitro.* Manimaran et al. (2014) successfully treated a patient suffering from ORN with the combination of allogeneic expanded DP-MSCs, tricalcium phosphate and PRP after conventional method failure. The 6-months follow-up was uneventful. A periodic orthopantogram (OPG) revealed appreciable bone formation (Manimaran et al., 2014). Two years later, a patient with an ameloblastoma was cared for with the combination of expanded autologous DP-MSCs, SVF, PRP, PRF, and βTCP. The authors observed no recurrence during the one and a half year follow-ups. Bone regeneration was confirmed through medical imaging (Manimaran et al., 2016). Since then, different approaches have been studied to improve bone regeneration using DP-MSCs, such as a specific scaffold (Petridis et al., 2015), the cell sheet technique (Fujii et al., 2018), and inflammation (Tomasello et al., 2017). However, most of the studies are preclinical studies. Petridis et al. (2015) observed bone formation in a rat calvarial defect model using human DPMSCs in a hydrogel scaffold. Tomasello et al. (2017) observed *in vitro* that inflamed dental tissue-derived MSCs showed a higher proliferative and osteogenic potential. Furthermore, inflammation increases several actin-depolymerizing factors (ADFs) and heat shock proteins (HSPs), playing a role in bone regeneration (Tomasello et al., 2017). To bypass the need for a scaffold, Fujii et al. (2018) regenerated bone through DPMSC cell sheet technology in association with a helioxanthin derivative *in vivo*.

The following section will describe MSC/EPC coculture in bone regeneration, benefits in angiogenesis and osteogenesis and preclinical applications.

## Contribution of MSC/EPC Coculture in Bone Regeneration

### Vascularization and EPCs

Successful bone regeneration requires neovascularization along with an efficient blood supply (Rouwkema and Khademhosseini, 2016; Wu et al., 2019). Neovascularization is achieved through vasculogenesis and angiogenesis. Vasculogenesis consists of *de novo* blood vessel formation by the differentiation and assembly of angioblastic progenitor cells during embryogenesis. Postnatal vasculogenesis, defined as the incorporation of circulating EPCs into the microvascular endothelium of newly developing microvessels, plays a major contribution to adult neovascularization. Angiogenesis consists of new blood vessels sprouting from the preexisting vasculature (Rouwkema and Khademhosseini, 2016; Wu et al., 2019).

First isolated by Asahara et al. (1997), EPCs are a population of unipotent progenitors displaying self-renewability, clonogenicity and differentiation capacity that can be easily isolated from peripheral blood-derived from several sources such as BM, spleen, umbilical cord, liver, kidney and other sources (Stolk and van der Geest, 1998; Goerke et al., 2015; Rana et al., 2018). EPCs stimulate angiogenesis and osteogenesis through soluble factor release (BMP-1, 2, 3, 6, 7, and 8, VEGF, TGF-β, etc.) and thus play a significant role in bone formation (Liu et al., 2012; Pang et al., 2013; Jia et al., 2019; Wu et al., 2019). Recent results have highlighted that EPCs increase angiogenesis and osteogenesis by incorporating themselves into newly formed blood vessels, mainly by recruiting resident MSCs and EPCs in the bone forming site (Tamari et al., 2020).

### Benefits of the Coculture

Coculture of MSCs and EPCs aims to obtain a synergistic effect in terms of angiogenesis and bone regeneration (Sun et al., 2016). Usami et al. (2009) observed that coculture of BM-MSC/peripheral blood mononuclear cell-EPCs (PBMC-EPCs) coculture is beneficial for bone tissue engineering. The authors highlighted that even if the soluble factors secreted by EPCs did not facilitate osteogenic differentiation, the newly formed vasculature may enhance bone regeneration. Greater bone formation, more mature trabecular bone formation, and higher neovascularization were highlighted in the coculture (Usami et al., 2009). Positive synergy between BM-MSCs and PBMC-EPCs *in vivo* translated into improved early vascularization followed by enhanced bone regeneration (Seebach et al., 2010; Zigdon-Giladi et al., 2015). Interestingly, Seebach et al. (2012) noticed in a BM-MSC/PBMC-EPC combination *in vivo* that EPCs improved vascularization in a bone defect directly through vessel formation and indirectly through the release of growth factors, such as VEGF, recruiting host EPCs. Geuze et al. (2009) observed, in an MSC/EPC coculture *in vitro*, that both cell types enhanced proliferation. However, BM-MSCs/BM-EPCs at different ratios (1/4;1/1;4/1) did not show a higher bone content than the monoculture of BM-MSCs when keeping the total numbers of seeded cells constant *in vivo* (Geuze et al., 2009). EPCs and MSCs can be cocultured *in vitro* on cancellous bone under osteogenic conditions. Early EPCs maintain endothelial differentiation. Coculturing PBMC-EPCs with BM-MSCs stabilizes the latter’s collagen-1α gene expression, which might be beneficial in bone healing (Henrich et al., 2013). Li and Wang (2013) observed that coculture of BM-MSCs and BM-EPCs in direct contact at a ratio of 1/1 can induce upregulation of angiogenic growth factors such as VEGF and IGF-1 and generate a favorable environment for angiogenesis, which in turn favors osteogenesis. 3D cell constructs can be fabricated *in vitro* by incorporating human umbilical vein endothelial cells (HUVECs) into MSC constructs, promoting the survival rate and osteogenic differentiation of MSCs (Sasaki et al., 2015). Through the use of 3D coculture systems, Genova et al. (2016) showed that endothelial cells (ECs) are able to stimulate the osteodifferentiation of MSCs, enhancing bone production. According to this study, a virtuous loop between MSCs and ECs seems central for the osteogenesis process (Genova et al., 2016). The study of Bidarra et al. (2011) demonstrated that by coculturing MSCs with HUVECs, there was not only an enhancement of osteogenic differentiation but also a significant increase in MSC proliferation. Li et al. (2014) demonstrated that a coculture of peripheral blood (PB)-CD34^+^ cells and BM-MSCs with HA could more efficiently promote bony regeneration than MSC composites alone in a model of a calvarial critical-size defect in rabbits. To recreate a biological “regenerative” microenvironment, the authors constructed cell sheets mixing MSCs and PB-CD34^+^. This technology has been proven to be effective for harvesting cells together with their endogenous extracellular matrix so that adhesion molecules on the surface of the cell and cell-cell interactions remain intact (Li et al., 2014). Liang et al. (2016) demonstrated that *in vitro* coculture of MSCs/EPCs, derived from BMNC, in cell sheets at a ratio of 1/1 promoted osteoblast differentiation. Real-time (RT) PCR revealed that osteogenic-associated gene (Runx2, OCN, and Col-I) and angiogenic-associated gene (VEGF-A and KDR) expression levels were significantly higher in EPC-MSC sheets. Furthermore, *in vivo* analyses confirmed bone healing regeneration. Quantitative analyses revealed more bone and vessels. The authors suggested that EPCs might provide a local environment favoring MSC osteogenic differentiation (Liang et al., 2016). Wen et al. (2016) established a BM-EPC/BM-MSC indirect coculture system in vitro and *in vivo*, allowing only soluble factor exchange. The authors demonstrated that BM-MSCs cocultured with BM-EPCs maintained their initial biological properties: no morphological changes, continuous expression of cell genes but negative expression of an endothelial phenotype and pericyte surface markers. Moreover, the authors noticed that the EPC microenvironment positively influenced the proliferative ability of MSCs. Furthermore, the expression of the pluripotency factors OCT4, SOX2, Nanog, and Klf4 (core regulators of cell stemness) was upregulated in coculture as much as the transcription of osteoblastic marker genes (OCN, BSP, and Runx2). EPCs have dynamic roles in maintaining MSC stemness and in regulating their differentiation potential. MSC and EPC combined with fibrin glue (FG) showed improved bone regeneration, with more bone and more cancellous bone with blood vessel structures, when used to repair rat alveolar bone defects compared to monoculture grafts *in vivo* (Wen et al., 2016).

### Preclinical Applications

Bone tissue engineering aims to replace the use of autologous bone grafts, the actual gold standard. Nau et al. (2018) demonstrated in an animal model that an induced membrane filled with BM-MNCs or EPCs derived from the spleen and MSCs seeded on βTCP supports bone defect healing to a similar degree as transplanted syngeneic bone. Thus, cell therapy approaches might be feasible to reduce the use of syngeneic bone grafts during the application of the induced membrane technique (Nau et al., 2018). Currently, their combination is studied in different clinical applications, such as reconstruction of bone defects after radiotherapy or maxillary sinus augmentation. BM-MSC/BM-EPC sheets promote bone healing in irradiated rats. BM-EPCs improved the osteogenic differentiation of BM-MSC sheets and enhanced ectopic bone formation (Liu et al., 2018). BM-MSC/BM-EPC coculture on bio-Oss significantly enhanced adhesion and ALP activity *in vitro*. Their association with maxillary sinus augmentation resulted in significantly greater bone formation (height, compressive strength, bone volume density, trabecular thickness, and trabecular number and a significantly lower trabecular separation) *in vivo* (Li et al., 2020).

### Sources of MSCs in Coculture

Besides, BM-MSC, AT-MSCs could be an interesting source of MSCs, considering their proangiogenic properties, high resistance to hypoxia, and ease of isolation (Wang et al., 2020). The AT-MSC/BM-EPC combination increases osteogenesis and angiogenesis-related gene expression *in vitro*. The results of animal experiments demonstrated that the AT-MSC/BM-EPC association accelerates critical-sized bone defect repair (He et al., 2020). Coculture of AT-MSC sheet-BM-EPCs generated more bone and cartilage. The newly formed tissue was denser, and the vascular lumen structures were more mature. Intramembranous and enchondral ossification processes coexist (Wang et al., 2020). Hayrapetyan et al. (2016) noticed that ECs showed a positive effect on osteogenic differentiation and mineralization in both direct and indirect culture systems in AT-MSC/HUVEC coculture. However, HUVECs had no effect on AT-MSCs proliferation. Direct coculture of AT-MSCs with HUVECs seems to be more effective than indirect coculture, probably due to simultaneous direct cell–cell contact and actions of secreted soluble molecules. AT-MSCs accounted for only 50% of the total cells in the AT-MSC/HUVEC coculture but achieved equal levels of mineralization compared to the AT-MSC monoculture (Hayrapetyan et al., 2016).

Endothelial progenitor cells and MSCs are thus commonly used to promote vessel formation and osteoblastic differentiation in tissue engineering. However, the underlying mechanisms of vessel formation and osteoblastic differentiation remain unclear. We will further describe the different interactions cell-cell, cell-matrix and cell-soluble factors coexisting between MSCs and EPCs.

## Interactions Between EPCS and MSCS in Coculture

**FIGURE 2:**
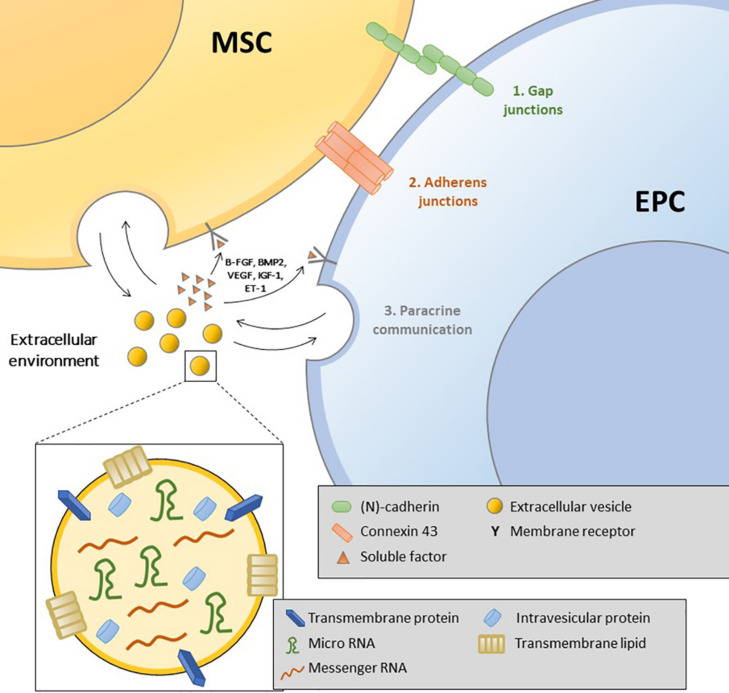
Illustration of the different types of interaction between mesenchymal stromal cells and endothelial progenitor cells: gap junctions, adherence junctions, soluble factors, and extracellular vesicles.

Bone formation requires intimate cooperation between osteoprogenitors and endothelial cells (Grellier et al., 2009b). Intercellular interactions include cell–cell, cell–matrix, and cell–soluble factors. Grellier et al. (2009a) suggested the coexistence of three communication pathways: gap junctions, adherence and tight junctions, and diffusible factor secretion activating specific receptors on target cells (Figure 2).

### Gap Junction Communication: The Role of Connexin 43 (Cx43)

Villars et al. (2000) showed that BM-MSCs express and synthesize VEGF. HUVEC-conditioned medium has a proliferative effect, and early osteoblastic marker levels increase when these cells are cocultured with HUVECs only in direct contact. Interestingly, the authors highlighted that differentiation was not modulated by VEGF alone. Direct contact between HUVECs and bone cell progenitors was mandatory (Villars et al., 2000). This signaling involves different heterotypic connections requiring adhesion molecules or gap junctions, such as Cx43 (Villars et al., 2002). Communication through Cx43 gap junctions increases the expression of early osteoblastic differentiation markers by osteoprogenitors. Cx43 expression in ECs and BM-MSCs contributes to the regulation of gene expression in osteoblastic cells, but the mechanisms remain elusive. Guillotin et al. (2004) reported that cell cooperation exists between ECs derived from large or small vessels and those derived from cord blood and osteoprogenitor cells. In accordance with their previous work, the authors suggested that ECs may support initial osteoblastic proliferation but do not alter the ability of osteoblasts to produce extracellular mineralizing matrix (ECM) (Guillotin et al., 2008). Direct contact during HUVEC/human osteoprogenitor (HOP) coculture results in cell rearrangement, giving rise to tubular-like networks promoted by soluble factors. VEGF alone could not affect EC migration under these conditions. However, VEGF seems to play some role in coculture osteoblastic differentiation (Grellier et al., 2009b).

### Adherence Junctions: Neural (*N*)-Cadherin, Expressed by Both Osteoblasts and ECs

(*N*)-cadherin is widely expressed in multiple tissues and participates in heterotypic contacts between different cell types. (*N*)-cadherin is expressed by both osteoblasts and ECs (Grellier et al., 2009a). Li et al. (2010) observed that (*N*)-cadherin expression was significantly upregulated in human BM-MSC and HUVEC cocultures. (*N*)-cadherin was concentrated in the cocultured human BM-MSC membrane but distributed within the cytoplasm of monocultured human BM-MSCs, indicating that cell–cell adhesion was improved in cocultured cells (Li et al., 2010). In addition, more beta-catenin was found to translocate into the cocultured cell nuclei, and more T cell factor-1 (TCF-1) was detected. Finally, the mRNA levels of early osteoblastic markers, including alkaline phosphatase (ALP) and type I collagen (Col-I), were significantly upregulated and subsequently increased (*N*)-cadherin expression. Interestingly, a link between (*N*)-cadherin and Cx43 has been suggested to underscore this effect (Bidarra et al., 2011).

### Paracrine Communication

Paracrine signaling requires diffusible factors spreading through the ECM. For Ern et al. (2010) the scarcity of MSC and EC contacts in a coculture suggests the influence of growth factor-mediated cell interactions. Wang et al. (1997) proposed bilateral osteoendothelial communication involving soluble factors such as VEGF. MSCs and ECs produce VEGF, which is able to modulate the growth and differentiation of both cell types (Wang et al., 1997; Mayer et al., 2005). Growth factors produced by ECs, including endothelin-1 (ET-1), insulin-like growth factor (IGF), and bone morphogenetic protein-2 (BMP-2), can modulate MSC osteogenic differentiation through interactions with specific membrane receptors (Grellier et al., 2009b). ET-1 is a vasoconstrictor secreted by ECs; it is involved in the regulation of craniofacial development and MSC osteogenic differentiation. Tsai et al. (2015) demonstrated that ECs regulate MSC functions through the secretion of ET-1 and AKT signaling. In response to VEGF, ECs can produce IGF-1, which in turn induces an upregulation of ALP in osteoblasts (Wang et al., 1997). The role of BMP-2 in coculture was highlighted by Kaigler et al. (2005). The inhibition of BMP-2 expression by ECs results in a significant decrease in osteogenic differentiation of BM-MSCs cocultured with ECs. bFGF can act on both ECs and MSCs, activating EC proliferation and inducing ALP and Col-1 in osteoprogenitors (Distler et al., 2003; Marie, 2003). These interactive effects may be mediated by the MAPK/ERK signaling pathway. The ECM components also support the interactions between ECs and osteoprogenitors. ECM produced by HUVECs increases the expression of ALP in osteoblasts (Lampert et al., 2016). TGF-β is expressed by ECs and osteoprogenitors and is sequestered in the ECM. After its release from the ECM, cell migration of both cell types is induced to recruit cells to the bone healing site (Wang et al., 2008).

### Extracellular Vesicles: A New Pathway of Communication

Extracellular vesicles released by almost any cell have an important role in cell-to-cell communication. EVs are generally classified into exosomes (EXOs), 30–100 nm in diameter, initially derived from endosomes as intraluminal vesicles, and microvesicles (MVs), 50–1000 nm in diameter, generated by outward budding and fission of the plasma membrane (van Niel et al., 2018). Recently, it has been proposed that the beneficial paracrine effects observed after MSC therapy might be mediated, at least in part, by EVs. EVs can be incorporated into cells via endocytosis or phagocytosis, leading to transfer of their contents (proteins, lipids, DNA, RNA, and mitochondria). Among these constituents, microRNAs are small non-coding RNA molecules that have a prominent role in gene regulation and biological functions. Both MSC-EV release and content are modified by environmental conditions (Gorgun et al., 2020). Xie et al. (2017) observed that MSC-derived EVs promote dose-dependent HUVEC proliferation, migration and tube formation. *In vitro* experiments showed that MSC-derived EVs had no major effect on the proliferation, apoptosis or osteogenesis of MSCs, indicating that EV-modified scaffolds promote bone regeneration mainly by accelerating vascularization (Xie et al., 2017). Qin and Zhang (2017) reported the EPC communication and regulation of BM-MSCs through EPC-derived EVs. In this study, EPC-EVs increased MSC proliferation and inhibited their osteoblastic differentiation (Qin and Zhang, 2017). In contrast, Jia et al. (2019) in an animal model, observed that EPC-EVs accelerate osteogenesis and bone consolidation during distraction osteogenesis by stimulating angiogenesis. More trabecular mature bone and less fibrous or cartilaginous tissues were discovered after EPC or EPC-EV treatment (Jia et al., 2019). Interestingly, EPC-EVs enhanced the proliferation, migration and angiogenic capacity of HUVECs through exosomal miR-126. In addition, EPC-Exos increase the expression of angiogenesis-related genes (VEGFa, bFGF, TGFβ1, and ANG) in HUVECs (Xu et al., 2018).

## Conclusion

Bone regeneration is a complex phenomenon involving a cell source, a scaffold, tissue-inducing factors (signaling factors) and mechanical stimulation. Preclinical and clinical studies have demonstrated that MSCs have added value in bone regeneration. Different sources of MSCs, expanded or not, are being explored. However, the great variability in terms of MSC source, dose, methodology and outcome measures renders direct comparison of the studies difficult, and studies require standardization.

Nevertheless, vascularization remains a key component. MSC and EPC coculture fosters not only osteogenesis but also angiogenesis with positive synergy. The EPCs enable and hasten osteogenesis. Knowing their precocious action in the differentiation process, EPCs are considered osteoinductive mediators. However, research still needs to optimize the knowledge of different elements, such as the cell population ratio. Cell-to-cell contact, ECM and soluble factors collaborate toward bone regeneration. Notwithstanding, the exact interaction mechanisms between these two cell populations remain unexplained. Recent studies have shown that the microenvironment participates in bone regeneration through EV production. The latter transport elements such as microRNAs, playing a key role in bone formation. Thorough studies should be conducted to better understand the role of each cell population and the nature of their interactions for bone regeneration.

## Author Contributions

CB, PP, DD, IL, DB, LL, and NM: conceptualization. CB, FC, and LL: writing. CB, PP, DD, IL, DB, LL, and NM: editing. All authors read and agreed to the published version of the manuscript.

## Conflict of Interest

The authors declare that the research was conducted in the absence of any commercial or financial relationships that could be construed as a potential conflict of interest.
